# Long-term prognostic value of thyroid hormone levels in chronic critical illness patients

**DOI:** 10.1080/07853890.2025.2479583

**Published:** 2025-03-21

**Authors:** Zhaoxiang Li, Liang Wang, Jianling Shi, Weiying Han, Chengrui Zhu, Tingrui Zhang, Xiaochun Ma, Yingjian Liang

**Affiliations:** ^a^ Department of Critical Care Medicine, The First Hospital of China Medical University, Shenyang, China; ^b^ Department of Critical Care Medicine, Tacheng District People’s Hospital, Tacheng, China

**Keywords:** Nonthyroidal illness syndrome, chronic critical illness, triiodothyronine, predictive model, prognosis

## Abstract

**Background:**

Chronic critical illness (CCI) can manifest as dysfunction of thyroid hormones. This study aimed to investigate the predictive value of the nonthyroidal illness syndrome (NTIS) for the prognosis of CCI patients, establish a predictive model for the prognosis of CCI patients, and evaluate the efficacy of the model to provide a theoretical basis for clinical intervention.

**Methods:**

This was a prospective observational study in which patients ≥18 years old who met the CCI criteria were enrolled. The primary outcome of the study was 90-day mortality after intensive care unit (ICU) admission. A nomogram was constructed to predict the prognosis of CCI patients, and the model was evaluated via the concordance index, calibration curve and decision curve analysis.

**Results:**

A total of 545 patients were included, and NTIS patients accounted for 65.3% of the patients. CCI patients with NTIS had more ventilator days and higher 90-day mortality. Lower free triiodothyronine (FT3) levels (<1.19 pmol/L) or reduced free thyroxine (FT_4_) levels (<9.655 pmol/L) were significantly associated with reduced survival in CCI patients with NTIS. Older age, a higher Sequential Organ Failure Assessment (SOFA) score, an emergency other than a traumatic operation, and a lower FT4 and thyroid-stimulating hormone level were found to be independent prognostic factors for a fatal outcome in CCI patients. The *C*-index for the prediction nomogram was 0.734, and the bias-corrected *C*-index was 0.727. The area under the receiver operating characteristic curve of our prediction model was superior to that of the SOFA and Acute Physiology and Chronic Health Evaluation II scores.

**Conclusions:**

Decreased serum FT3 and FT4 concentrations in patients with CCI at admission to the ICU on day 10 are associated with 90-day mortality. Early detection of serum FT3 and FT4 levels could help clinicians target CCI patients at high risk of clinical deterioration.

## Introduction

Thyroid hormones play crucial roles in regulating thermoregulation, energy expenditure and cellular metabolism [1]. In critical illness, the most common alterations are low plasma concentrations of triiodothyronine (T3), low or normal plasma concentrations of thyroxine (T4), or elevated plasma rT3 in the presence of normal thyroid-stimulating hormone (TSH). Together, these changes differ from those in primary and secondary thyroid disorders, which are referred to as nonthyroidal illness syndrome (NTIS) [2,3]. NTIS may cause severe disease-induced injury that is closely related to the prognosis of critically ill patients. A meta-analysis including 6869 critically ill patients from 25 studies revealed that NTIS was independently associated with an increased risk of death in critically ill patients [4]. When disseminated intravascular coagulation caused by sepsis is complicated with NTIS, patients with this condition have significantly greater severity and a higher rate of mortality [5].

Advancements in modern intensive care technology have enabled more critically ill patients to survive. However, there is also a subset of patients whose condition progresses from an acute to a chronic state. These patients often have residual organ dysfunction and require ongoing life-sustaining interventions. Those who remain in the intensive care unit (ICU) for extended periods and are susceptible to recurrent infections, persistent chronic inflammation, acquired immune suppression and protein catabolism are diagnosed with chronic critical illness (CCI). These patients are characterized by a long hospital stay, multiple organ disorders, a high mortality rate, and considerable consumption of resources [6]. CCI patients exhibit suppressed neuroendocrine function with low circulating levels of several anterior pituitary-dependent hormones, including low activity of the thyroid stimulating axis [7,8]. The development of NTIS in CCI patients seems to be a consequence of severe illness and injury. Compared with non-NTIS patients, patients with NTIS have worse ICU outcomes, as illustrated by impaired cardiac function, diaphragmatic dysfunction, an inability to be weaned off the ventilator and a longer ICU duration [9].

Although some studies have suggested that NTIS is associated with poor patient prognosis, most studies have been conducted on general ICU populations or specific patient groups with certain diseases [10–15]. There are few studies on the prognosis of CCI patients with NTIS; in particular, the value of the NTIS for the prediction of long-term outcomes in CCI patients has not been reported. Therefore, we conducted a prospective observational study to provide both prognostic information for patients and guidance for future interventional research in this CCI population.

## Materials and methods

### Design and subjects

This prospective, observational study involved adult patients admitted to the ICU of the First Affiliated Hospital, China Medical University, from 1 April 2017 to 31 October 2023. Adult patients (≥18 years) with a CCI were enrolled. The diagnostic criteria for CCI were as follows: stayed in the ICU for eight days or more and had one or more of the following five states: mechanical ventilation lasting more than 96 h without breaks, tracheostomy, sepsis/severe infections, serious wounds or multiple organ dysfunction syndrome [16]. Patients were excluded if they had (1) a diagnosis of any thyroid disease, such as hyperthyroidism, hypothyroidism or thyroid tumours; (2) a history of thyroidectomy; (3) a pregnancy within the previous 6 months; or (4) a history of dopamine, glucocorticoid or thyroid hormone therapy. The study was approved by the Ethics Committee of the First Hospital of China Medical University (No. 2016-166-2) and was carried out in accordance with the Declaration of Helsinki. Owing to the effects of sedatives and analgesics, the majority of the patients in our study were unconscious and unable to provide informed consent. Informed consent was signed by the unconscious patients’ family members and conscious patients.

### Data collection and follow-up

The data describing clinical features and laboratory parameters were collected on the 10th day after ICU admission [17,18]. Information was collected from the electronic medical records and included demographic information (i.e. age, sex), the worst Acute Physiology and Chronic Health Evaluation II (APACHE II) score, the worst Sequential Organ Failure Assessment (SOFA) score, the sources of patients admitted to the ICU, underlying conditions, laboratory parameters and outcomes. The laboratory parameters included absolute lymphocyte count, C-reactive protein (CRP), procalcitonin (PCT), CD4^+^ T cells, CD8^+^ T cells, complement protein 3 (C3), complement protein 4 (C4), albumin, prealbumin and transferrin.

All thyroid hormone levels were determined on days 10, 17, 24 and 31 after ICU admission. Serum free triiodothyronine (FT3), free thyroxine (FT4) and TSH were measured via chemiluminescent immunoassays. The reference values in our laboratory are as follows: FT3: 2.43–6.01 pmol/L, FT4: 9.01–19.05 pmol/L and TSH: 0.35–4.94 mIU/L. The diagnostic criteria for NTIS are FT3 < 2.43 pmol/L with FT4 < 9.01 pmol/L or normal, and TSH < 0.35 mIU/L or normal. The included patients were evaluated in each time point, and divided into two groups: the NTIS group and the non-NTIS group. The primary outcome observed in this study was 90-day mortality after admission to the ICU. The secondary outcomes of this study included 30-day mortality, hospital length of stay (LOS), ICU LOS and duration of mechanical ventilation. The critically ill patients who discontinued therapy and were automatically discharged were followed-up with phone calls to confirm their clinical outcomes. Only the first admission was included if the patient had multiple admissions during the study period.

### Statistical analysis

Multiple imputation (MI) was used to account for missing data if the missing values were less than 20%, and variables with a missing rate of more than 20% were excluded. MI was performed via Bayesian methods in SPSS (SPSS Inc., Chicago, IL). The data were tested via the Kolmogorov–Smirnov normality test and Bartlett’s test for homogeneity of variance. The data are presented as counts (%) for categorical variables and as the means ± standard deviations or medians (25th–75th percentiles) for continuous variables, as appropriate. The Mann–Whitney *U*-test, Fisher’s exact test and Chi-square analysis were used to test for differences between groups as appropriate. Kaplan–Meier’s plots with log-rank statistics were used to assess differences in survival between the NTIS group and the non-NTIS group. Univariate analyses for 90-day mortality were performed through Cox regression. Multicollinearity was evaluated using the variance inflation factor (VIF), with a threshold of 10 used to identify highly correlated variables. The variables selected were subsequently subjected to multivariate Cox regression analysis, and a forward stepwise method was used to determine the independent predictors. Independent predictors of mortality according to the multivariate Cox regression analysis were applied to construct a prediction model, and a nomogram was constructed to visualize the model. The discrimination ability of the nomogram was evaluated via the concordance index (*C*-index). To account for bias and variability, we employed 1000 iterations of bootstrap resampling to internally calibrate the *C*-index. A calibration curve was used to assess the concordance between the nomogram-predicted probability and the actual probability. Decision curve analysis (DCA) was used to evaluate the clinical utility of the nomogram by quantifying the net benefits at different threshold probabilities. A two-sided *p* value <.05 was considered to indicate statistical significance. All the data were analysed via IBM SPSS Statistical version 25.0 (SPSS, Chicago, IL) and R software 4.1.1 (R Foundation for Statistical Computing, Vienna, Austria).

## Results

### Baseline characteristics of the study samples

**Figure 1.**
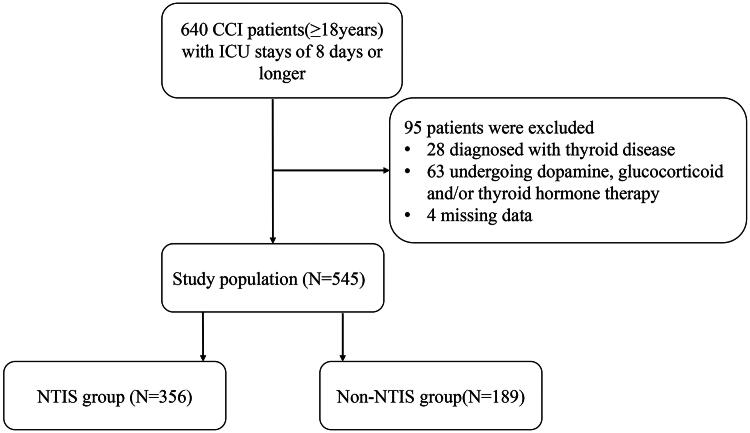
Flowchart of the study cohort. ICU: intensive care unit; CCI: chronic critical illness; NTIS: nonthyroidal illness syndrome.

In total, 640 adult patients were identified with the inclusion criteria. We excluded 28 patients who were diagnosed with thyroid disease or who underwent thyroidectomy; 63 patients who received glucocorticoid, dopamine or thyroid hormone therapy; and four patients whose data were missing. Therefore, 545 patients were enrolled and followed-up in this study (Figure 1). The baseline demographic characteristics and laboratory examination results of 545 individuals in the study population are shown in Table 1. In our study, NTIS accounted for 65.3% of all the patients. The median age was 66 (25th–75th percentile, 54–78) years, and 242 (68.0%) patients were men. Age, sex, admission type (expect medical) and underlying disease status (except for chronic liver failure and malignant tumours) were not significantly different between the NTIS group and the non-NTIS group. Patients in the medical ICU had a greater risk for NTIS than non-NTIS patients did. Among the two groups, NTIS patients were more likely to have greater chronic liver failure and malignant tumour history; APACHE II score; SOFA score; CRP; and PCT but had lower absolute lymphocyte counts; CD4^+^ T cells; CD8^+^ T cells; and C3, C4, albumin, prealbumin, transferrin, FT3, FT4 and TSH levels. Additionally, substantial differences in clinical outcomes were observed between the two groups. Compared with patients without NTIS, patients with NTIS had more ventilator days (*p* < .001), greater 30-day mortality (*p* < .001) and greater 90-day mortality (*p* < .001). There was no significant difference in the length of hospital stay (*p* = .542) or duration of ICU stay (*p* = .183) between the two groups (Table 1).

**Table 1.** Baseline clinical and laboratory characteristics of study subjects.

	All (*n* = 545)	Non-NTIS group (*n* = 189)	NTIS group (*n* = 356)	*p* Value
Age(years), median (p25–p75)	66 (53, 78)	66 (52, 79)	66 (54, 78)	.845
Female/male, *n*	168/377	54/135	114/242	.406
Admission type				
Elective operation, *n* (%)	81 (14.9)	23 (12.2)	58 (16.3)	.198
Medical, *n* (%)	241 (44.2)	99 (52.4)	142 (39.9)	.005
Trauma, *n* (%)	63 (11.6)	22 (11.6)	41 (11.5)	.966
Emergency surgery other than trauma, *n* (%)	160 (29.4)	45 (23.8)	115 (32.3)	.038
Underlying condition				
Hypertension, *n* (%)	204 (37.4)	76 (40.2)	128 (36.0)	.328
Chronic heart failure, *n* (%)	135 (24.8)	48 (25.4)	87 (24.4)	.805
COPD, *n* (%)	30 (5.5)	12 (6.3)	18 (5.1)	.557
Chronic renal failure, *n* (%)	33 (6.1)	10 (5.3)	23 (6.5)	.707
Chronic liver disease, *n* (%)	12 (2.2)	0 (0)	12 (3.4)	.010
Malignant tumour, *n* (%)	57 (10.5)	10 (5.3)	47 (13.2)	.004
Immunosuppression, *n* (%)	15 (2.8)	4 (2.1)	11 (3.1)	.593
Diabetes mellitus, *n* (%)	114 (20.9)	32 (16.9)	82 (23.0)	.095
APACHE II, median (p25–p75)	16 (13, 20)	14 (11, 17)	16.0 (13, 20)	<.001
SOFA, median (p25–p75)	6 (3, 8)	4 (3, 6)	6.5 (4, 9)	<.001
Absolute lymphocyte count, median × 10^9^/L (p25–p75)	0.97 (0.68, 1.40)	1.05 (0.75, 1.47)	0.94 (0.65, 1.37)	.034
CRP (mg/L), median (p25–p75)	88.50 (45.40, 145.00)	57.70 (23.80, 111.50)	105.50 (59.85, 164.45)	<.001
PCT (ng/mL), median (p25–p75)	0.509 (0.200, 2.000)	0.238 (0.130, 0.590)	0.845 (0.280, 3.115)	<.001
CD3^+^ T cells (cells/μL), median (p25–p75)	613.0 (412.0, 866.0)	701.0 (477.0, 946.0)	567.5 (388.0, 811.5)	<.001
CD4^+^ T cells (cells/μL), median (p25–p75)	385.0 (252.0, 558.0)	449.0 (278.0, 594.0)	357.5 (228.0, 521.5)	<.001
CD8^+^ T cells (cells/μL), median (p25–p75)	188.0 (123.0, 324.0)	227.0 (142.0, 418.0)	173.5 (117.0, 291.5)	<.001
C3 (g/L), median (p25–p75)	1.01 (0.76, 1.25)	1.14 (0.90, 1.36)	0.94 (0.68, 1.18)	<.001
C4 (g/L), median (p25–p75)	0.23 (0.16, 0.30)	0.26 (0.20, 0.32)	0.21 (0.14, 0.28)	<.001
Albumin (g/L), median (p25–p75)	25.50 (22.70, 28.40)	27.00 (24.40, 30.30)	24.55 (21.70, 27.30)	<.001
Prealbumin (mg/dl), median (p25–p75)	10.7 (7.5, 15.1)	12.9 (9.2, 17.1)	9.8 (6.7, 13.9)	<.001
Transferrin (mg/dl), median (p25–p75)	113.0 (88.4, 141.0)	128.0 (103.0, 164.0)	107.5 (82.1, 131.0)	<.001
Thyroid function				
FT3 (pmol/L), median (p25–p75)	2.14 (1.60, 2.61)	2.86 (2.59, 3.16)	1.80 (0.82, 2.13)	<.001
FT4 (pmol/L), median (p25–p75)	10.96 (9.03, 12.72)	12.41 (11.29, 14.06)	10.12 (8.12, 11.62)	<.001
TSH (mIU/L), median (p25–p75)	1.34 (0.53, 2.34)	1.68 (0.92, 2.70)	1.12 (0.37, 2.19)	<.001
30-day mortality, *n* (%)	134 (24.6)	24 (12.7)	110 (30.9)	<.001
90-day mortality, *n* (%)	199 (36.5)	48 (25.4)	151 (42.4)	<.001
Ventilator days (days), median (p25–p75)	10 (7, 16)	8 (5, 14)	11 (8, 18)	<.001
Duration of ICU stay (days), median (p25–p75)	16 (12, 27)	15 (12, 26)	17 (12, 28)	.183
Length of hospital stay (days), median (p25–p75)	34 (21, 16)	30 (20, 53)	35 (22, 54)	.542

ICU: intensive care unit; COPD: chronic obstructive pulmonary disease; APACHE II: Acute Physiology and Chronic Health Evaluation; SOFA: Sequential Organ Failure Assessment; CRP: C-reactive protein; PCT: procalcitonin; C3: complement protein 3; C4: complement protein 4; FT3: free triiodothyronine; FT4: free thyroxine; TSH: thyroid-stimulating hormone; p25–p75: percentile 25th–75th.

### Thyroid hormone levels and outcomes

**Figure 2.**
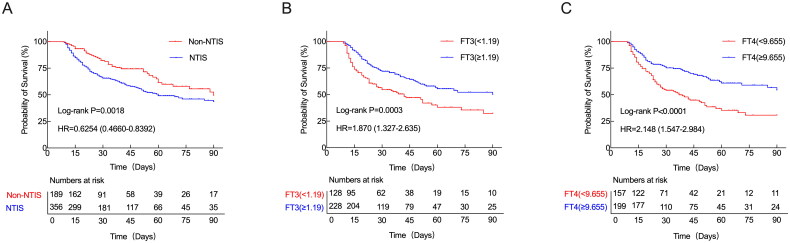
Survival curves of CCI patients. (A) Kaplan–Meier’s survival curves of CCI patients with NTIS and non-NTIS; Kaplan–Meier’s survival curves of CCI patients with NTIS based on the FT3 cutoff value of 1.19 pmol/L (B) and the FT4 cutoff value of 9.655 pmol/L (C) on day 10. A log-rank test was used to evaluate differences between groups. CCI: chronic critical illness; FT3: free triiodothyronine; FT4: free thyroxine.

**Figure 3.**
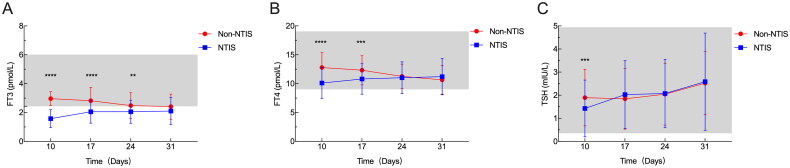
Dynamic changes in FT3, FT4 and TSH levels in CCI patients. The lowest FT3 (A), FT4 (B) and TSH (C) levels were observed on day 10 after admission to the ICU within the 10–31-day range. After day 10, the serum FT3, FT4 and TSH levels in the NTIS group gradually increased. However, the serum FT3 level was still significantly lower in the NTIS group than in the non-NTIS group on days 17 and 24, and the serum FT4 level was still significantly lower in the NTIS group than in the non-NTIS group on day 17. CCI: chronic critical illness; NTIS: nonthyroidal illness syndrome; TSH: thyroid-stimulating hormone; FT4: free thyroxine; FT3: free triiodothyronine. **p < .01; ***p < .001; ****p < .0001.

Survival analysis of patients with NTIS compared with patients without NTIS was performed. Among all the CCI patients, the NTIS was significantly associated with poorer 90-day survival (*p* < .001) according to the Kaplan–Meier curves (Figure 2(A)). We also found that in CCI patients with NTIS, lower FT3 levels (<1.19 pmol/L) or reduced FT4 levels (<9.655 pmol/L) were significantly associated with reduced survival (Figure 2(B,C)). From day 10 onward, the serum FT_3_, FT_4_ and TSH levels were substantially lower in the NTIS patients than in the non-NTIS patients (*p* values: *p* < .0001, *p* < .0001 and *p* < .001, respectively). After 10 day, the serum FT3, FT4 and TSH levels in the NTIS group gradually increased. However, the serum FT3 level was still significantly lower in the NTIS group than in the non-NTIS group on days 17 and 24, and the serum FT4 level was still significantly lower in the NTIS group than in the non-NTIS group on day 17 (Figure 3(A–C)). The majority of NTIS patients had TSH levels within the normal range.

### Construction of the predictive model

Univariate and multivariate Cox regression analyses of the associations between each indicator and 30-day and 90-day mortality are summarized in Table 2 and Supplementary Tables 1 and 2. The variables significantly related to 90-day mortality were age; trauma patients; emergency surgery other than trauma patients; the APACHE II score; SOFA score; chronic heart failure; chronic liver disease; immunosuppression; CRP; PCT; CD3^+^; CD4^+^; CD8+ T cells; C3; C4; albumin; prealbumin; transferrin; FT3; FT4; and TSH. CD3^+^ T cell VIF >10 indicates highly multicollinearity with other variables and was not included in the multivariate Cox regression analysis. The remaining variables were included in the forward stepwise multivariate Cox regression analysis to determine the independent predictors of 90-day mortality. As shown in Table 3, advanced age (*p* < .001), a high SOFA score (*p* < .001), emergency surgery other than trauma (*p* < .001), a low FT4 level (*p* = .003) and a low TSH level (*p* = .031) were found to be independent prognostic factors for fatal outcomes in CCI patients.

**Table 2.** Univariable cox regression analyses for 90-day mortality.

	*β*	Wald *χ*^2^	*p* Value	HR	95%CI	
					Lower limit	Upper limit
Age	0.015	12.296	<.001	1.015	1.006	1.023
Sex (male)^a^	0.098	0.391	.532	1.103	0.812	1.497
Admission type						
Elective operation	−0.374	2.740	.098	0.688	0.442	1.071
Trauma	−0.719	6.280	.012	0.487	0.278	0.855
Emergency surgery other than trauma	0.408	7.599	.006	1.504	1.125	2.010
Medical	0.062	0.189	.664	1.064	0.805	1.405
Underlying condition						
Hypertension	0.080	0.307	.580	1.084	0.816	1.439
Chronic heart failure	0.444	8.611	.003	1.558	1.159	2.096
COPD	0.302	1.183	.277	1.352	0.785	2.328
Chronic renal failure	0.472	3.456	.063	1.602	0.975	2.635
Chronic liver disease	0.870	5.108	.024	2.388	1.123	5.079
Malignant tumour	0.064	0.081	.777	1.066	0.685	1.661
Immunosuppression	0.782	5.798	.016	2.186	1.157	4.130
Diabetes mellitus	−0.092	0.277	.599	0.912	0.647	1.286
APACHE II score	0.084	67.524	<.001	1.088	1.066	1.110
SOFA score	0.196	114.535	<.001	1.216	1.173	1.260
Absolute lymphocyte count	−0.042	0.236	.627	0.959	0.809	1.137
CRP	0.005	29.269	<.001	1.005	1.003	1.006
PCT	0.026	39.526	<.001	1.027	1.018	1.035
CD3^+^ T cells	−0.001	12.236	<.001	999	0.999	1.000
CD4^+^ T cells	−0.001	12.306	<.001	0.999	0.998	1.000
CD8^+^ T cells	−0.001	4.473	.034	0.999	0.997	1.000
C3	−1.108	27.306	<.001	0.33	0.218	0.500
C4	−2.768	13.997	<.001	0.063	0.015	0.268
Albumin	−0.061	13.623	<.001	0.941	0.911	0.972
Prealbumin	−0.048	13.116	<.001	0.953	0.929	0.978
Transferrin	−0.009	20.707	<.001	0.-991	0.988	0.995
Thyroid function						
FT3	−0.491	34.095	<.001	0.612	0.519	0.722
FT4	−0.151	31.027	<.001	0.860	0.816	0.907
TSH	−0.237	13.642	<.001	0.789	0.696	0.895

COPD: chronic obstructive pulmonary disease; APACHE II: Acute Physiology and Chronic Health Evaluation; SOFA: Sequential Organ Failure Assessment; CRP: C-reactive protein; PCT: procalcitonin; C3: complement protein 3; C4: complement protein 4; FT3: free triiodothyronine; FT4: free thyroxine; TSH: thyroid-stimulating hormone; HR: hazard ratio; CI: confidential interval.

^a^ Female as reference.

**Table 3.** Multivariable Cox regression analyses for 90-day mortality.

	*β*	Wald *χ*^2^	*p* Value	HR	95%CI	
					Lower limit	Upper limit
Age	0.016	13.448	<.001	1.016	1.008	1.025
Emergency surgery other than trauma	0.487	10.458	.001	1.628	1.212	2.187
SOFA score	0.175	74.495	<.001	1.189	1.143	1.237
FT4	−0.085	8.834	.003	0.918	0.869	0.971
TSH	−0.133	4.418	.036	0.875	0.773	0.991

SOFA: Sequential Organ Failure Assessment; FT4: free thyroxine; TSH: thyroid-stimulating hormone.

**Figure 4.**
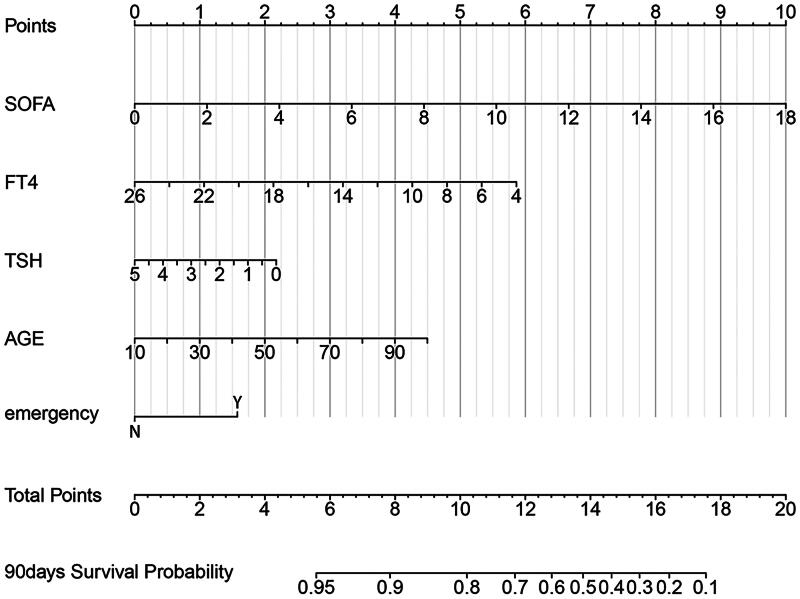
Nomogram for predicting the 90-day survival probability in CCI patients. SOFA: Sequential Organ Failure Assessment; TSH: thyroid-stimulating hormone; FT4: free thyroxine; N: no; Y: yes.

On the basis of the five independent predictors, a predictive model was constructed to predict mortality outcomes in CCI patients and was visualized via a nomogram (Figure 4). Each predictive factor was assigned a single score, which is presented on the top line of the nomogram. The total score of each patient is the sum of each single score. At the bottom of the nomogram, the probabilities of hospital mortality in CCI patients with NTIS were predicted in terms of the total scores.

### Evaluation and validation of the prediction model

**Figure 5.**
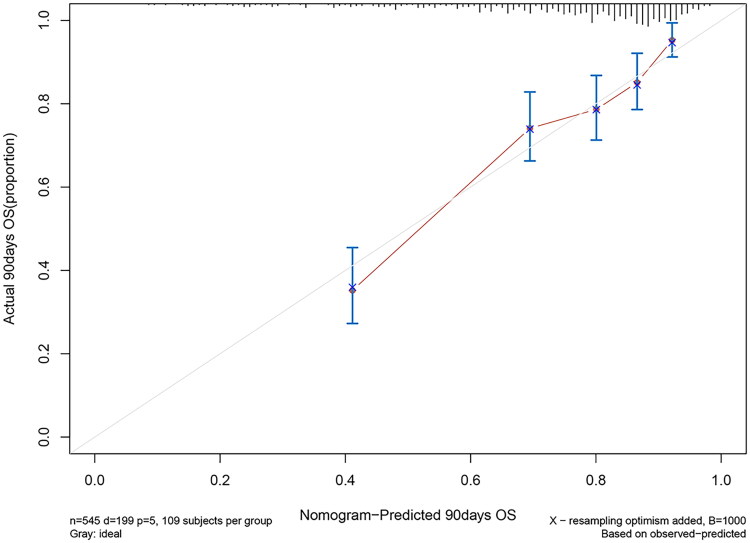
Calibration curves of the nomogram. OS: overall survival. *n* = 370, *d* = 99, *p* = 4; 74 subjects per group. Grey: ideal. X – resampling optimism added, *B* = 1000. On the basis of the observed predictions.

**Figure 6.**
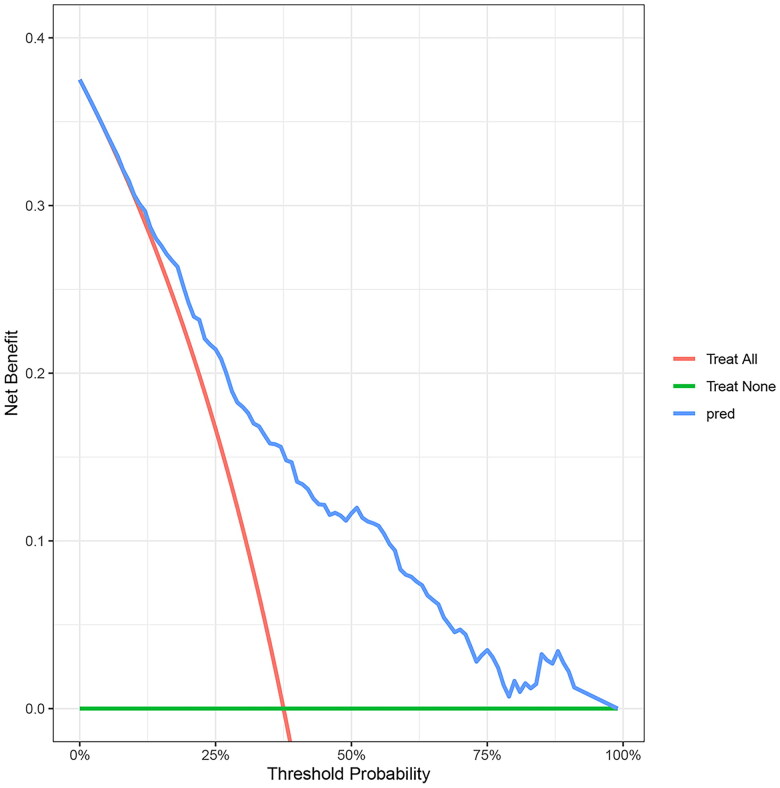
DCA of the nomogram. DCA: decision curve analysis.

Using the bootstrap method, the *C*-index for the prediction nomogram was 0.734, suggesting proper discrimination by the model. The bias-corrected *C*-index was 0.727. The calibration curve did not significantly deviate from the reference line (Figure 5). The nomogram showed good consistency between the 90-day OS prediction and the actual 90-day OS observation. Subsequently, DCA was performed to evaluate the clinical applicability of the prediction model. As shown in Figure 6, DCA demonstrated that the nomogram had superior overall net benefits compared to the ‘treat all’ and ‘treat none’ strategies when the threshold probability ranged from 13% to 100%, with the highest net benefit reaching 0.29. This indicates high potential for clinical utility within this threshold probability range.

**Figure 7.**
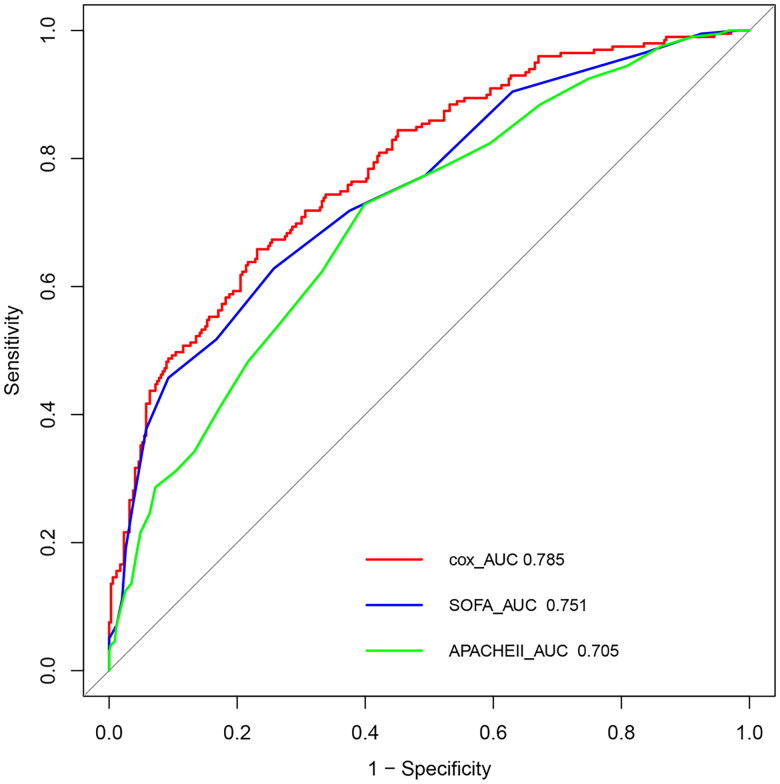
A ROC curve of our prediction model, the SOFA score and the APACHE II score. SOFA: Sequential Organ Failure Assessment; APACHE: Acute Physiology and Chronic Health Evaluation.

We performed receiver operating characteristic (ROC) curve analysis to compare the predictive accuracy of the SOFA and APACHE II scores for 90-day mortality. The area under the receiver operating characteristic curve (AUROC) of our prediction model was 0.785, which was greater than the AUC for the SOFA score (AUC 0.751) and the AUC for the APACHE II score (AUC 0.705) (Figure 7).

## Discussion

In our study, we found that CCI patients with NTIS had more ventilator days and higher 30-day and 90-day mortality rates. Lower FT3 levels (<1.19 pmol/L) or reduced FT4 levels (<9.655 pmol/L) were significantly associated with reduced survival in CCI patients with NTIS. Older age, a higher SOFA score, an emergency other than a traumatic operation, and a lower FT4 and TSH level were found to be independent prognostic factors for a fatal outcome in CCI patients. The *C*-index for the prediction nomogram was 0.734, and the bias-corrected *C*-index was 0.727. The calibration curve and DCA also demonstrated its accuracy and applicability. The AUROC of our prediction model was superior to that of the SOFA and APACHE II scores. Therefore, early identification of high-risk individuals among CCI patients for personalized treatment is crucial for improving patient prognosis.

The characteristics of CCI patients can be summarized as neuroendocrine dysfunction, organ dysfunction or immune dysfunction, which can manifest as dysfunction of thyroid hormones. Several studies have demonstrated that thyroid hormones are associated with prognosis in patients with critical illnesses. Low T3/FT3 is commonly observed in these patients. In septic shock patients, a decrease in the serum level of T3 is associated with ICU mortality [15]. Additionally, in non-selected ICU patients, FT3 remains a valid independent predictor of ICU mortality [14]. A low serum FT3 concentration not only affects the short-term prognosis of critically ill patients but also affects the long-term prognosis [19]. In the prolonged phase of critical illness, a concomitant and gradual decrease in the serum T4 and TSH levels may also occur [20]. The decrease of T4/FT4 and TSH in NTIS is a marker of disease severity [21,22]. Both the database from the Medical Information Mart for Intensive Care (MIMIC)-III and study on paediatric patients with meningococcal septic shock suggest that low T4/FT4 is an independent predictor of an unfavourable outcome [23,24]. When T3 and T4 levels both decrease, the patient’s prognosis worsens, and the mortality rate significantly increases [25]. In a study evaluating neonates with sepsis in ICU, lower baseline TSH is associated with higher mortality [26]. In our study, we found that lower FT4 and TSH levels were independent risk factors for 90-day mortality in CCI patients with NTIS. Subgroup analysis revealed that when FT3 was less than 1.19 pmol/L and FT4 was less than 9.655 pmol/L, the 90-day survival rate of CCI patients was lower, which is consistent with the view that the more severe the thyroid hormone changes were, the worse the prognosis of critically ill patients [27].

In clinical work, some prognostic scores are needed to estimate mortality and disease severity. At present, the APACHE II and SOFA scores are well established and commonly used in the ICU, but both mainly evaluates the acute phase of the disease and are not suitable for chronic severe patients; therefore, we developed a new predictive model to predict 90-day mortality in CCI patients. When we evaluate the performance of a prediction model, we can have indicators such as sensitivity, specificity and AUC, but they are all indicators of accuracy, and cannot tell us whether the model is worth using, that is, to solve the problem of clinical value of the model. DCA is a new way to evaluate predictive models, it is essentially decision analysis, so it can decide whether to use a model, or which of several models is optimal [28,29]. The AUC of our prediction model was 0.785, which was superior to those of the SOFA score (AUC 0.751) and the APACHE II score (AUC 0.705), which was consistent with previous research [30]. DCA demonstrated that our predictive model had good overall net benefits within a wide range of threshold probabilities.

There were several limitations in our study. First, we conducted an observational study that did not allow us to draw certain conclusions leading to therapeutic interventions. Second, the data were obtained from a single centre. This study lacked an external validation cohort to validate the predictive ability of the nomogram; however, whether the prediction model is applicable to other populations is obscure. Therefore, it is necessary to assess its potential by testing the model in different and larger populations. Moreover, further multicentre prospective studies are needed to verify the results.

These findings suggest that decreased serum FT3 and FT4 concentrations in patients with CCI at admission to the ICU on day 10 are associated with 90-day mortality. Early detection of serum FT3 and FT4 levels could help clinicians target CCI patients at high risk of clinical deterioration.

## Supplementary Material

Supplemental Material

## Data Availability

The datasets used and/or analysed during the current study are available from the corresponding author on reasonable request.
