# Supplementary material for: Long-term prognostic value of thyroid hormone levels in chronic critical illness patients
Source: Ann Med. 2025 Mar 21;57(1):2479583. doi: 10.1080/07853890.2025.2479583 (PMC11934158; doi:10.1080/07853890.2025.2479583)
Supplement: Supplemental Material [file IANN_A_2479583_SM1214.zip › supplementary_file/Supplement Table 1.docx]

Supplement table 1, Univariable Cox regression analyses for 30-day mortality

|  | β | Wald χ2 | P value | HR | 95%CI | |
| --- | --- | --- | --- | --- | --- | --- |
|  |  |  |  |  | lower limit | upper limit |
| Age | 0.006 | 1.309 | 0.253 | 1.006 | 0.996 | 1.015 |
| Sex (Male)* | 0.169 | 0.747 | 0.387 | 1.184 | 0.808 | 1.734 |
| Admission type |  |  |  |  |  |  |
| Elective operation | -0.109 | 0.193 | 0.661 | 0.897 | 0.552 | 1.457 |
| Trauma | -0.503 | 2.339 | 0.126 | 0.605 | 0.318 | 1.152 |
| Emergency surgery other than trauma | 0.582 | 10.889 | 0.001 | 1.789 | 1.266 | 2.527 |
| Medical | -0.298 | 2.795 | 0.095 | 0.743 | 0.524 | 1.053 |
| Underlying condition |  |  |  |  |  |  |
| Hypertension | -0.148 | 0.661 | 0.416 | 0.862 | 0.604 | 1.232 |
| Chronic heart disease | 0.127 | 0.432 | 0.511 | 1.135 | 0.777 | 1.658 |
| COPD | 0.477 | 2.295 | 0.130 | 1.612 | 0.869 | 2.988 |
| Chronic renal failure | 0.429 | 1.854 | 0.173 | 1.535 | 0.828 | 2.846 |
| Chronic liver disease | 0.948 | 5.149 | 0.023 | 2.581 | 1.138 | 5.855 |
| Malignant tumor | 0.206 | 0.628 | 0.428 | 1.228 | 0.739 | 2.043 |
| Immunosuppression | 0.555 | 1.762 | 0.184 | 1.741 | 0.768 | 3.949 |
| Diabetes mellitus | -0.455 | 3.666 | 0.056 | 0.634 | 0.398 | 1.011 |
| APACHE II score | 0.081 | 43.34 | <0.001 | 1.085 | 1.059 | 1.111 |
| SOFA score | 0.195 | 80.471 | <0.001 | 1.216 | 1.165 | 1.269 |
| Absolute Lymphocyte Count | -0.029 | 0.079 | 0.778 | 0.972 | 0.795 | 1.187 |
| CRP | 0.005 | 28.866 | <0.001 | 1.005 | 1.003 | 1.007 |
| PCT | 0.027 | 41.008 | <0.001 | 1.028 | 1.019 | 1.036 |
| CD3+ T cells | -0.001 | 10.385 | 0.001 | 0.999 | 0.999 | 1.000 |
| CD4+ T cells | -0.001 | 12.637 | <0.001 | 0.999 | 0.998 | 0.999 |
| CD8+ T cells | -0.001 | 3.730 | 0.053 | 0.999 | 0.998 | 1.000 |
| C3 | -1.016 | 15.317 | <0.001 | 0.362 | 0.218 | 0.602 |
| C4 | -2.519 | 7.686 | 0.006 | 0.081 | 0.014 | 0.478 |
| Albumin | -0.068 | 11.163 | 0.001 | 0.934 | 0.898 | 0.972 |
| Prealbumin | -0.065 | 13.795 | <0.001 | 0.937 | 0.906 | 0.970 |
| Transferrin | -0.010 | 16.538 | <0.001 | 0.991 | 0.986 | 0.995 |
| Thyroid function |  |  |  |  |  |  |
| FT3 | -0.630 | 34.982 | <0.001 | 0.533 | 0.432 | 0.656 |
| FT4 | -0.187 | 29.889 | <0.001 | 0.830 | 0.776 | 0.887 |
| TSH | -0.380 | 19.800 | <0.001 | 0.684 | 0.579 | 0.809 |

COPD Chronic Obstructive Pulmonary Disease, APACHE II Acute Physiology and Chronic Health Evaluation, SOFA Sequential Organ Failure Assessment, CRP C-reactive protein, PCT procalcitonin, C3 Complement protein 3, C4 Complement protein 4, FT3 free triiodothyronine, FT4 free thyroxine, TSH thyroid-stimulating hormone, HR hazard ratio, CI confidential interval. * Female as reference.
