# Supplementary material for: Long-term prognostic value of thyroid hormone levels in chronic critical illness patients
Source: Ann Med. 2025 Mar 21;57(1):2479583. doi: 10.1080/07853890.2025.2479583 (PMC11934158; doi:10.1080/07853890.2025.2479583)
Supplement: Supplemental Material [file IANN_A_2479583_SM1214.zip › supplementary_file/Supplement Table 2.docx]

Supplement table 2, Multivariable Cox regression analyses for 30-day mortality

|  | β | Wald χ2 | P value | HR | 95%CI | |
| --- | --- | --- | --- | --- | --- | --- |
|  |  |  |  |  | lower limit | upper limit |
| Emergency surgery other than trauma | 0.641 | 13.070 | <0.001 | 1.899 | 1.341 | 2.688 |
| SOFA score | 0.174 | 51.401 | <0.001 | 1.190 | 1.135 | 1.248 |
| FT3 | -3.111 | 7.463 | 0.006 | 0.733 | 0.587 | 0.916 |
| TSH | -0.281 | 11.685 | 0.001 | 0.755 | 0.643 | 0.887 |

SOFA Sequential Organ Failure Assessment, FT3 free triiodothyronine, TSH thyroid-stimulating hormone
